# A Systematic, Integrated Study on the Neuroprotective Effects of Hydroxysafflor Yellow A Revealed by ^1^H NMR-Based Metabonomics and the NF-**κ**B Pathway

**DOI:** 10.1155/2013/147362

**Published:** 2013-04-22

**Authors:** Yuanyan Liu, Zeqin Lian, Haibo Zhu, Yinghong Wang, Shishan Yu, Tingting Chen, Jing Qu, Jianbei Li, Shuanggang Ma, Xianhong Chen

**Affiliations:** ^1^State Key Laboratory of Bioactive Substance and Function of Natural Medicines, Institute of Materia Medica, Chinese Academy of Medical Sciences and Peking Union Medical College, Beijing 100050, China; ^2^School of Chinese Materia Medica, Beijing University of Chinese Medicine, Beijing 100102, China

## Abstract

Hydroxysafflor yellow A (HSYA) is the main active component of the Chinese herb *Carthamus tinctorius* L.. Purified HSYA is used as a neuroprotective agent to prevent cerebral ischemia. Injectable safflor yellow (50 mg, containing 35 mg HSYA) is widely used to treat patients with ischemic cardiocerebrovascular disease. However, it is unknown how HSYA exerts a protective effect on cerebral ischemia at the molecular level. A systematical integrated study, including histopathological examination, neurological evaluation, blood-brain barrier (BBB), metabonomics, and the nuclear factor-κB (NF-κB) pathway, was applied to elucidate the pathophysiological mechanisms of HSYA neuroprotection at the molecular level. HSYA could travel across the BBB, significantly reducing the infarct volume and improving the neurological functions of rats with ischemia. Treatment with HSYA could lead to relative corrections of the impaired metabolic pathways through energy metabolism disruption, excitatory amino acid toxicity, oxidative stress, and membrane disruption revealed by ^1^H NMR-based metabonomics. Meanwhile, HSYA treatment inhibits the NF-κB pathway via suppressing proinflammatory cytokine expression and p65 translocation and binding activity while upregulating an anti-inflammatory cytokine.

## 1. Introduction

Stroke is one of the leading causes of adult disability and death in developing countries. Vascular cognitive impairment is recognized as a widespread and preventable syndrome [1]. With the increasingly rapid aging process, cerebrovascular disease, such as stroke, is a very important public health and societal problem. The flower of the safflower plant, *Carthamus tinctorius* L., and its extracts have been extensively used in traditional Chinese medicine for the treatment of cerebrovascular and cardiovascular diseases [2]. Previous work has indicated that HSYA, the main chemical component of the safflower yellow pigment, can antagonize binding of the platelet activating factor to its receptor [3], produce antihypotensive and antithrombotic effects, inhibit platelet aggregation [4], and exhibit neuroprotective effects after permanent middle cerebral artery occlusion (pMCAO) in rats [5, 6]. Injectable safflor yellow (50 mg, containing 35 mg HSYA) has been widely used in Chinese medicine, and it was approved as a new drug by the State Food and Drug Administration (SFDA) for treating patients with ischemic cardiocerebrovascular disease in 2005. Moreover, a second clinical study of HSYA has been approved by the China SFDA for the treatment of diseases of blood vessels in the brain. The subchronic toxicity study of HSYA indicated that it is generally well tolerated [7].

In cerebral ischemia, there is ample evidence for the activation of the NF-**κ**B [8, 9]. NF-**κ**B induces the expression of both proinflammatory genes and genes related to apoptosis [10]. In peripheral cells, NF-**κ**B is crucial for inflammation reactions [11] but increases excitotoxic damage in the hippocampus [12]. Although it has been established that inflammation contributes to cerebral ischemic injury and that HSYA is an effective neuroprotectant, it is not known how HSYA exerts a protective effect at the molecular level and if it can travel across the BBB. It is also not known if NF-**κ**B is altered or if the inflammatory factors are affected in order to achieve neuroprotection.

Metabonomics is a well-established field in systems biology [13] and has been applied to observe meaningful and relevant biochemical changes due to disease, toxicity, nutrition, and other variables [14]. It is an important methodology for measuring the relative concentrations of endogenous small molecules in biofluids and characterizing changes in the metabolites in organisms [15]. Nuclear magnetic resonance spectroscopy (NMR) and high-performance liquid chromatography coupled with multistage tandem mass spectrometry (HPLC-MS^n^) have been used to study such changes, allowing for metabolite screening to be performed over a wide range of concentration [9, 13]. Metabonomics studies can provide invaluable information towards understanding molecular mechanisms and novel insights into the status of dysfunction in biological systems.

In this study, the effect of HSYA on the infarct volume and neurological score of rats with focal cerebral ischemic injury was investigated. Specifically, an HPLC-MS^n^ method was developed for detecting HSYA transport across the BBB in different rat brain tissues after intravenous injection. Furthermore, a metabonomics method was employed to characterize the metabolic profile associated with MCAO-induced cerebral ischemia and observe the protective effects of HSYA in brain tissue. The metabolic disturbance was studied using high-resolution magic-angle spinning (HR MAS) NMR spectroscopy combined with pattern recognition methods. Based on the previous studies, the role of HSYA in the NF-**κ**B pathway after pMCAO was investigated.

## 2. Materials and Methods

### 2.1. Animals

 Male Sprague-Dawley (SD) rats, 12 weeks old, weighing 280–290 g were housed in an environmentally controlled room and food and water ad libitum. The rats underwent permanent MCAO as described in Supplementary Materials (MCAO model construction) available online at http://dx.doi.org/10.1155/2013/147362 [16]. All protocols in this study were approved by the Medical Ethic Committee of Peking Union Medical College and were in accordance with National Institutes of Health regulations for the care and use of animals in research.

### 2.2. Drug Administration

The *Carthamus tinctorius* samples were purchased from the Beijing Tongrentang Medicine Corporation Ltd. (Beijing, China). HSYA was isolated and purified in our laboratory and confirmed by HRMS^n^ and ^1^H, ^13^C NMR (purity > 98%, HPLC). HSYA was dissolved in normal saline and administrated intravenously (i.v.) at doses of 10 mg/kg (pretreated), 10 mg/kg (posttreated), 50 mg/kg (posttreated), and 10 mg/kg (5 times posttreated). Pyrrolidine dithiocarbamate ammonium salt (PDTC, Sigma), a potent NF-**κ**B suppressor [17], followed the same dosage regimen as HSYA to be compared to each other at equal pace. Normal control and the sham-operation animals received vehicle in the same manner as the drug-treated group.

### 2.3. Samples

After animal sacrificed, brains were rapidly removed and immersed in chilled artificial cerebrospinal fluid (118 mM NaCl, 4.8 mM KCl, 2.0 mM CaCl_2_, 1.2 mM MgSO_4_, 25.0 mM NaHCO_3_, and 12.0 mM KH_2_PO_4_). The brain was sectioned into 3 mm coronal slices and targeted brain regions were dissected. All samples were snap-frozen in liquid nitrogen and stored at −80°C. The remaining tissues from the coronal slices were stored in formalin solution (0.026 mM NaH_2_PO_4_, 0.042 mM Na_2_HPO_4_, 1.322 mM NaCl, and 9% formaldehyde) for histologic stained.

20–50 mg frozen tissue samples was homogenized at 1000 rpm in 6 min in mixed solvent (CH_3_CN : H_2_O = 1 : 1). The supernatants were removed and dried under a stream of nitrogen before lyophilizing.

### 2.4. HPLE-MS^n^ Analyses

An Agilent 1100 Series liquid chromatography system was utilized. HPLC separation was carried on an Agilent Zorbax SB-C_18_ column (2.1 × 150 mm, 5 *μ*m) with a Phenomenex C_18_ guard column (4 × 20 mm, 5 *μ*m); the column was set at 35°C. The mobile phase was acetonitrile, 0.1% formic acid water (8 : 92, v/v). The flow rate was 0.4 mL/min, and the effluent was monitored at 275, 320, and 400 nm.

The ESI-MS^n^ experiment was performed on an Agilent 1100 Series LC/MSD Trap mass spectrometer (Santa Clara, CA, USA). The ESI conditions were as follows: HV capillary voltage 3.5 kV, drying temperature 350°C, drying gas (N_2_) 9.0 L/min, nebulizer gas (N_2_) 50 psi, and capillary exit voltage −124.8 V (negative). The function of smart fragmentation was set on (Smart Frag Ampl was 30%–200%).

### 2.5. ^1^H NMR Spectroscopy

Lyophilized brain tissue extracts weighing 40–80 *μ*g were reconstituted in 50 *μ*L of D_2_O containing 0.1% sodium 3-trimethylsilyl-2,2,3,3-d_4_-propionate (TSP, an internal standard, **δ** 0.0 ppm), and 40 *μ*L of each supernatant was transferred to narrow-mouth 4 mm rotors (Varian Inc., America). ^1^H-NMR spectra of liver tissues were recorded using the Carr-Purcell-Meiboom-Gill (CPMG) pulse sequence, with 256 transients, a spin-echo delay (*τ*) of 400 *μ*s, and a total spin-spin relaxation delay (2n*τ*) of 100 ms. The data were acquired with 128 scans and a spectral width of 8000 Hz and digitized into 64 K data points by a nano probe on a Varian INOVA-500 spectrometer (Varian Inc., America) operating at a proton frequency of 500.13 MHz. The samples were spun at 2-3 KHz and maintained at 300 K throughout the experiment. The FIDs were weighted by an exponential function with a 0.3 Hz line-broadening factor prior to Fourier transformation. Spectra were manually rephrased, and the baseline was corrected before reducing the data.

The biochemical data were expressed as the mean ± SD of five rats per group. Statistical analysis was performed using a one-way ANOVA. A calculated *P* value of less than 0.05 was considered statistically significant. 

### 2.6. Data Analysis

The acquired NMR spectra were referenced to the chemical shift of TSP. Following phase and baseline correction, the ^1^H-NMR spectra were automatically reduced to ASCII files using VNMR software (Varian, Inc.). The spectra were divided into 800 segments, each of 0.005 ppm wide, over a spectral window ranging from 0.5 to 4.5 ppm. The generated ASCII files were imported into Microsoft EXCEL for the addition of labels and then imported into SIMCA-P12.0 (Umetrics, Umeå, Sweden) for the pattern recognition analysis. Prior to the analysis, the values of all variables were centered and scaled.

Partial least square discriminant analysis (PLS-DA) was used to find differential metabolites between groups. PLS-DA is a frequently used PLS-based classification method where the response variable is a categorical one (dummy variables describing the categories) expressing the class membership of the statistical units. PLS-DA aims to find the best possible discriminant function (model) that separates classes of observations based on their *X* variables. When group separation was not satisfied based on PLS-DA, the data were further preprocessed using orthogonal partial least-squares discriminant analysis (OPLS-DA) to remove linear combinations of variables *X* that were orthogonal to the *Y* vector of the dependent variables, to eliminate the intersubject variability and describe maximum separation based on class [18]. Two-dimensional score plots are proved to be an efficient means of visualizing classification of the samples and investigating regions of the spectra that were altered as a result of compound dosing. The corresponding loading plots were used to identify which spectral variables contribute to the positioning of samples on the score plot and hence the variables that influence any observed separation in the data set.

### 2.7. Measurement of Infarct Size and Neurological Function

After 24 h of pMCAO, the neurological deficit score of each rat was obtained according to Longa's et al. method [16]. The neurologic findings were scored on a five-point scale: a score of 0 indicated no neurologic deficit, a score of 1 (failure to extend left forepaw fully) a mild focal neurologic deficit, a score of 2 (circling to the left) a moderate focal neurologic deficit, and a score of 3 (falling to the left) a severe focal deficit; rats with a score of 4 did not walk spontaneously and had a depressed level of consciousness. The brain slices were stained with a 2% solution of 2,3,5-triphenyl tetrazolium chloride (TTC) (Sigma) in saline at 37°C for 30 min and photographed. The volume of infarction was calculated according to the following formula *V* = ∑_*i*=1_^*n*−1^((*A*_*i*_ + *A*_*i*+1_)/2*h*), where *V* is the volume of fraction; *A*_*i*_ is the infarct area of each slice; and *h* is slice thickness.

### 2.8. Western Blot Analysis

Tissues were prepared as described in a previous report [19]. The blocked membranes were incubated overnight at 4°C with primary antibodies (1 : 400 dilution) against NF-**κ**B p65 or phospho-I**κ**B-**α**(Santa). Following 1 h secondary antibody (1 : 5000 dilution) incubation, the immunodetection was carried out by using enhanced chemiluminescence detection reagents. **β**-action was used as a loading control.

### 2.9. RNA Isolation and Semiquantitative NF-**κ**B p65, IL-1**β*,* and IL-10 RT-PCR

Cerebral cortex RNA extraction was prepared by using Trizol reagent (Invitrogen). Equal aliquots of the cDNA were amplified by PCR using specific primers (provided in supplemental Table 1S) at different cycles (30 cycles for p65, 35 cycles for IL-1*β* and IL-10, and 23 cycles for glyceraldehyde-3-phosphate dehydrogenase (GAPDH)). PCR products were then separated using a 1.5% agarose gel by gel electrophoresis, and band intensity was semiquantified by densitometry using GeneTools (Syngene) gel analysis software which was normalized by using GAPDH as an endogenous reference.

### 2.10. Nuclear Factor Binding Assay

NF-**κ**B DNA binding activity was assessed using Trans-AM transcription factor assay kits (Active Motif) according to the manufacturer's instructions. Five or ten micrograms of brain tissue nuclear extracts were added to 96-well plates. Antibody binding was measured using a luminometer. The specificity of nuclear factors activation was determined by competition experiments using wild-type and mutant consensus oligonucleotides provided with the kit. 

### 2.11. Statistical Analysis

All the results were obtained by a single experimenter, who was blinded to the experimental treatment groups and indicated at least three independent experiments expressing as mean ± SD. The unpaired *t*-test was performed to evaluate statistically significant differences; statistical significance was set at *P* < 0.05.

## 3. Results and Discussion

### 3.1. Effect of HSYA on the Infarct Volume and Neurological Score

**Figure 1:**
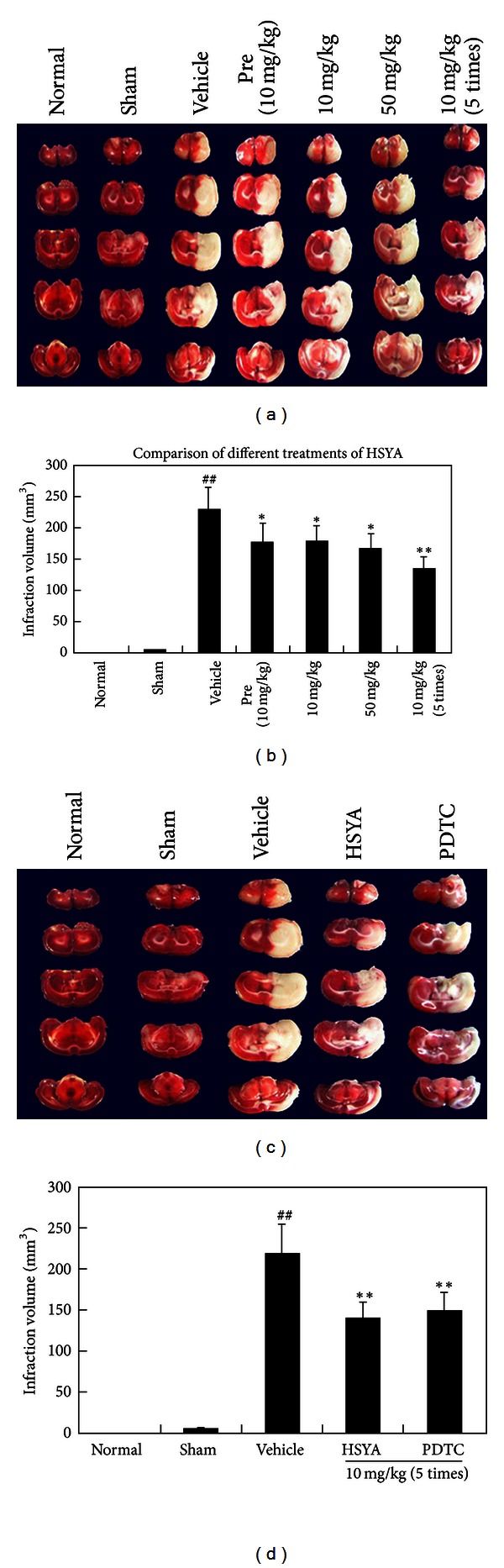
Effect of different administrations of HSYA and PDTC on infarct volume. (a, b) different administrations of HSYA; (c, d) HSYA and PDTC. A well-defined pale area was considered to be the infarct in the right hemisphere representative of TTC-stained sections after 24 h of pMCAO. ^##^ *P* < 0.01 compared with sham group; *P < 0.05, **P < 0.01 compared with vehicle-treated pMACO group.

TTC staining was used to evaluate the volume of infarction to compare the effect of different administrations of HSYA on pMCAO. As shown in Figures 1(a) and 1(b), the normal and sham-operated rats did not show any lesions in either hemisphere. However, 24 h after pMCAO, there was significant infarction (230 ± 20 mm^3^), as shown in the TTC-stained coronal brain section. Different treatments of HSYA, 10 mg/kg (pretreated), 10 mg/kg (posttreated), 50 mg/kg (posttreated), and 10 mg/kg (5 times posttreated), decreased the infarct volume by 21.3% (*P* < 0.05), 20.4% (*P* < 0.05), 21.5% (*P* < 0.05), and 30.9% (*P* < 0.01), respectively, compared to vehicle-treated MCAO rats. Successive administration of HSYA decreased the infarct volume more significantly than a single injection. Therefore, primarily successive administration of HSYA was used in subsequent experiments.

Meanwhile, the time point (24 h) and dosage regimen (i.v. successive administration, 10 mg/kg/30 min, 5 times) were determined; the effect of HSYA and PDTC on the infarct volume and neurological score after pMCAO was observed. As shown in Figures 1(c) and 1(d), normal and sham-operated rats did not show any lesions in either hemisphere, while 24 h after pMCAO, there was significant infarction (230 ± 20 mm^3^) in TTC-stained coronal brain sections. A neurological deficit score of 8.16 ± 0.75 was observed in vehicle-treated MCAO rats (provided in supplemental Table 2S). Clearly, HSYA and PDTC treatment decreased the infarct volume by 30.9% and 28.4%, respectively, and produced significant improvements in neurological functions (*P* < 0.01) compared to vehicle-treated MCAO rats. The effect of HSYA and PDTC on the infarct volume and neurological score did not show a significant difference when compared to each other.

### 3.2. Detection of HSYA in Different Rat Brain Tissues

**Figure 2:**
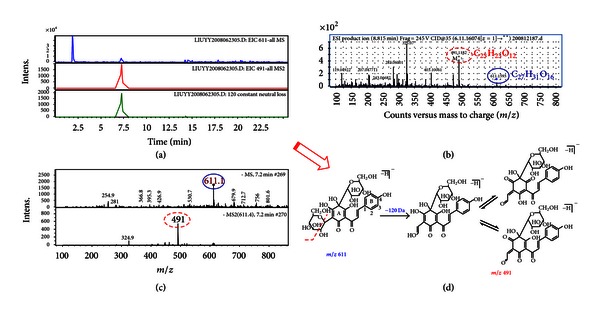
(a) HPLC/DAD/ESI-MS^n^ analyses of sample solution; (b) HPLC/ESI-MS^n^ spectra of [M–H]^−^ (*m/z* 611) ion in negative ion mode; (c) HPLC/HRMS data of [M–H]^−^ ion and its product ions for HSYA; (d) proposed fragmentation pathways for HSYA based on HPLC/HRMS data (*m/z* 611→491).

HSYA was detected in different rat brain tissues using HPLC/ESI-MS^n^ and HPLC/HRMS^n^ methods. The relationship between the structural characteristics and fragmentation behavior was investigated for HSYA. The HPLC/ESI-MS^n^ method is a reliable means of characterizing HSYA even in minute quantities. In addition, HPLC/HRMS^n^ was applied to verify the proposed fragmentation pathways. As shown in Figures 2(a)–2(c), HSYA with a retention time of 7.2 min was detected at *m/z *611 [M–H]^−^ in HPLC/(–)ESI-MS^n^ and *m/z *611.1593 in HPLC/HRMS^n^, suggesting a molecular weight of 612 Da and a molecular formula of C_27_H_31_O_16_. In the product ion mass spectra of [M–H]^−^, a prominent ion at *m/z *491 (i.e., 611–120) was observed in the MS^2^ spectrum. Furthermore, a peak at *m/z *491.1182 in HPLC/HRMS^2^ suggested a molecular formula of C_23_H_23_O_12_ and indicated that this was a diagnostic ion of HSYA. Accordingly, the proposed fragmentation pathways of HSYA are shown in Figure 2(d). HSYA was able to be unambiguously identified in extracts or biopsies based on its retention time, characteristic [M–H]^−^ ion at *m/z *611, fragment ion at *m/z *491, and the constant neutral loss of 120 Da. The limit of detection was 0.87 ng (signal/noise = 3.6). This validated method was applied to detect HSYA in different brain tissues (brain stem, cortex, hippocampus, and cerebellum) after i.v. administration of HSYA to vehicle-treated MCAO rats and normal rats. It was found that HSYA could be detected in each of the four brain tissues both for ischemic and normal groups.

### 3.3. ^1^H MAS NMR Metabonomics and Multivariate Statistical Analysis

In the previous studies, HSYA was verified to travel across the BBB and exhibit a protective effect on cerebral ischemia according to histopathological examination and neurological evaluation. Next, we examined the influence of HSYA on the endogenous small molecules in brain tissue. Here, we evaluated the beneficial effects of HSYA using ^1^H NMR-based metabonomics at a molecular level in brain samples.

**Figure 3:**
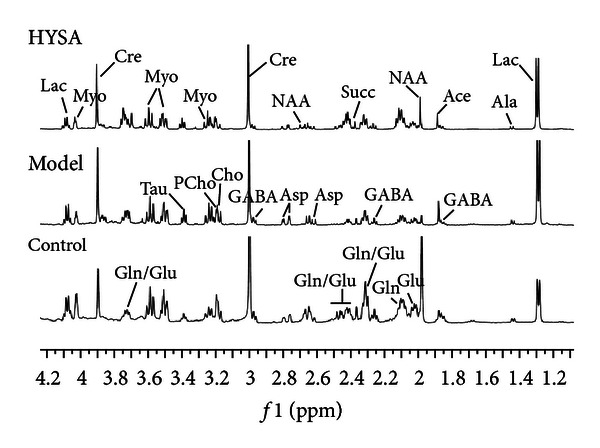
Typical 500 MHz ^1^H NMR CPMG spectra of brain tissue samples.

Figure 3 shows low-frequency regions of typical CPMG spectra of the polar metabolites extracted from rat brain tissues of control, ischemia and HSYA-treated rats. Based on literature reports [20, 21] and using the Chenomx NMR Suite software (Chenomx, Calgary, Canada), the major metabolites in brain tissues were identified, as shown in Figure 3 and Table 3S. Furthermore, a multivariate statistical method was applied to analyze the spectra more comprehensively and to identify metabolite signals that could efficiently differentiate between the treatment groups.

**Figure 4:**
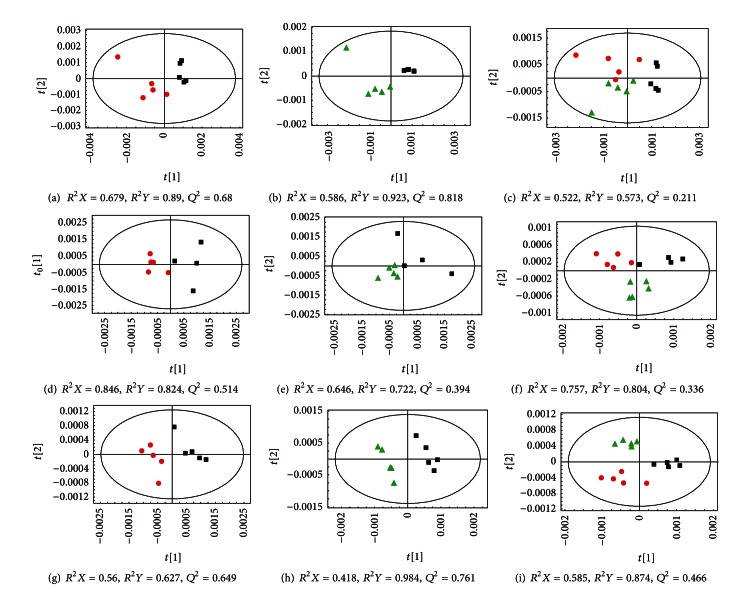
OPLS-DA scores plots from the analyses of the processed NMR spectra. (a–c) cerebellum, (d–f) cortex, and (g–i) hippocampus tissue extracts derived from three groups of rats: red circle, control; black square, ischemia; green triangle, HSYA.

We analyzed the metabolic variations in extracts of different brain tissues, including the cerebellum, cortex, hippocampus, and stem, after permanent focal cerebral ischemia using ^1^H-NMR spectroscopy and a multivariate statistical analysis. The OPLS-DA statistical analysis demonstrated that there were significant differences between the metabolic profiles of ischemia and control rats in the cerebellum, cortex, and hippocampus samples (Figures 4(a), 4(d), and 4(g)). The group that was administered HSYA deviated from the model group (Figures 4(b), 4(e), and 4(h)) and approached the control group, showing a trend of metabolic recovery (Figures 4(c), 4(f), and 4(i)). The metabolic profiles of the stem samples of ischemic and control animals were mostly the same, as the stem is a region of the brain that is resistant to hypoxia/ischemia [22]. Multivariate statistical algorithms were used to classify ^1^H NMR spectra of the brain cerebellum, cortex, and hippocampus in rats and identify distinct metabolic profiles for the three different regions. Loading plots from the OPLS-DA of the NMR spectra are provided in the supplementary materials Figure 1S.

The OPLS-DA scores and loading plot of the ^1^H-NMR data of cerebellum samples of ischemic rats showed an apparent higher level of lactate (Lac), alanine (Ala), choline (Cho), and phosphocholine (Pcho) along with a lower level of *N*-acetyl aspartate (NAA), Myo-inositol (Myo), creatine (Cre), and taurine (Tau) than in control rats. The model had an overall goodness of fit, *R* ^2^ *Y*, of 89% and an overall cross-validation coefficient, *Q* ^2^, of 68%. Treating ischemic animals with HSYA reverted some of the ischemic effects on the metabolite concentrations in the OPLS-DA loading plot. Compared with the ischemia group, the levels of Lac, Ala, Cho, and Pcho in the treatment group were significantly reduced and the levels of NAA, Cre, Myo, and Tau remarkably increased.

The predominant changes identified in the OPLS-DA analysis of cortex samples from ischemic rats included an increase in the signal intensities of Lac, acetate (Ace), Ala, glutamate (Glu), and aspartate (Asp), accompanied by a reduction in the intensities of NAA, Myo, Cre, Tau, and Gamma-aminobutyric acid (GABA). A general comparison between HSYA-treated and ischemic rats using an OPLS-DA model resulted in a clear differentiation between the two groups. The loading plot showed a decrease in Lac, Ace, Glu, and Asp and an increase in NAA, Cre, Myo, Tau, and GABA with treatment.

The metabolic changes in the hippocampus included a significant increase in the signal intensities of Lac, Ace, Cre, Glu, Glu/Gln, and Ala, accompanied by signal reductions of NAA, Tau, and GABA in ischemic animals. Moreover, significant differences were observed with HSYA treatment. Compared to ischemic rats without HSYA treatment, Lac, Ace, Glu, and Ala were remarkably reduced and NAA, Tau, and GABA were significantly increased in the treatment group as seen in the OPLS-DA loading plot.

**Figure 5:**
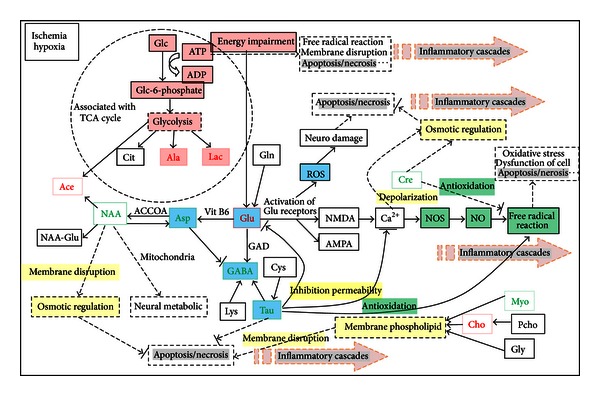
Summary of the metabolic pathways in which the metabolites described in this paper are involved. Red indicates metabolites with increased levels due to cerebral ischemia. Black indicates metabolites not found or not identified. Green indicates lowered metabolites due to cerebral ischemia. The pathways associated with energy metabolism, excitotoxicity, oxidative stress, and membrane disruption are labeled in red, blue, green, and yellow background, respectively.

Ischemic stroke causes a significant amount of cell damage resulting from an insufficient supply of glucose and oxygen to central nervous system (CNS) tissue. Lac is often used as an indicator of cerebral anoxia or hypoxia [23]. Overflow metabolism results in an incomplete oxidation of glucose, leading to the accumulation of Lac, Ace, Ala, and Glu and other incompletely oxidized metabolites associated with the glycolytic pathway [24]. They are the products of glycolysis and increase rapidly during hypoxia and ischemia to accommodate the extra energy demand required during ischemia [25]. The cells of the ischemic core undergo anoxic depolarization and are destined to die due to lack of energy [26]. As the number of depolarization increases, the infarcts grow larger [27]. As shown in Figure 5, a network map identified the tricarboxylic acid (TCA) cycle as playing a central role in the proposed signaling pathway that also interacts with numerous other pathways. Interestingly, the level of Lac in ischemic rats after treatment with HSYA significantly decreased, returning toward the normal level. This indicates that HSYA can modulate the changes in energy metabolism induced by ischemia and even reduce the infarct volume.

Following cerebral ischemia, there is an excessive release of Glu [28] and breakdown of astrocytic functions [29] that results in impaired Glu uptake by astrocytes [30]. Glu uptake reduces the glucose supply and exacerbates the energy impairment but can also lead to excitotoxicity [31]. Glu excitotoxicity is a significant determinant of ischemia pathophysiology. The increased levels of extracellular Glu most likely contribute directly to cell damage by calcium overload through excessive NMDA receptor activation or indirectly through initiating cascades such as free radical formation (Figure 5) [32]. An increase in the amount of oxygen free radicals leads to prompt dysfunction of the cellular membrane, resulting in necrosis [33]. Many studies have showed that the decreased Glu release might contribute to the neuroprotective effect of HYSA [34]. Spontaneous Glu release during ischemia could lead to excitotoxicity and perturbation of neural network functions [35].

On the contrary, Tau has been suggested to be a neuroprotective chemical [36], and its effects include calcium modulation, apoptosis inhibition, and protection against oxidative stress via its antioxidant properties [37]. Tau has also been implicated in the mechanism of cell shrinkage during apoptosis in several cell types, including cerebellar granule neurons [38]. Cell shrinkage is a distinctive characteristic of apoptotic cells. It is thought that changes in ion channel fluxes play a major role in this shrinkage [39]. Therefore, decreased Tau in the cerebellum and cortex of ischemic rats may not only indicate some deleterious information but also reflect part of the adaptive measures taken by the CNS. 

GABA, a key mediator of inhibitory neurotransmission in the mammalian central nervous system, is generated from Glu in GABAergic neurons by glutamic acid decarboxylase [40]. It is worth noting that this involves converting the principal excitatory neurotransmitter (Glu) into the principal inhibitory neurotransmitter (GABA). GABAergic agents protect against the delayed death of the CA1 hippocampal neurons elicited by ischemia. The changes in GABA during and after ischemia are sufficient to cause CNS depression or excitation [41]. In addition, Tau and lysine can increase both GABA synthesis and effects, while Asp and Glu most likely inhibit GABA effects [42]. In the present study, increased inhibitory neurotransmitter levels such as GABA and Tau accompanied with decreased excitatory neurotransmitter levels of Glu and Asp are associated with a better outcome of stroke when treated with HSYA.

Cerebral ischemia in particular is responsible for oxidative stress due to the generation of free radicals [43], which result in deleterious effects during pathogenesis [44]. Cre and Tau possess antioxidant properties in the brain [45]. Cre is thought to have a multifaceted role in the brain. Apart from being involved in brain osmoregulation, it has recently been implicated in energy homeostasis and protection from oxidative stress through acting directly as an antioxidant [46]. In the present study, Cre increased markedly in the hippocampus but decreased in the cerebellum and cortex of ischemic rats. An increase in Cre may reflect a protective mechanism against the enhanced oxidation, whereas a decline may indicate the exhaustion of Cre and lack of antioxidation in certain brain regions of ischemic rats. HSYA intervention could relatively elevate the levels of these two antioxidative markers.

NAA, a metabolite synthesized in the mitochondria, is commonly used as a marker of neuronal viability [47]. Extracellular NAA is transported into astrocytes [48] where it is rapidly hydrolyzed into Ace and Asp [49]. Considerable evidence shows that NAA has multiple roles in neurons, including neural metabolic function in mitochondria, osmoregulation, and axon-glial signaling [50, 51]. The reduction in NAA in a visible infarct was related to the reduction in blood flow to the infarct, which in turn was related to the infarct extent and clinical outcome [52]. Notably, NAA levels have clearly recovered after HSYA treatment, indicating that the disturbance of neuronal activity can be modified by HSYA.

Myo is a significant intracellular osmolyte, whose change may indicate fluctuations in tissue osmolarity [47]. It has been reported that Pcho, Myo, and glycerol are precursors used for the synthesis of membrane phospholipids in the cell and their levels play a role in lipid metabolism [47, 53]. For example, Pcho contributes to the Cho resonance, which may act as a biomarker for membrane phospholipid metabolism [54]. Therefore, changes in Pcho, Cho, and Myo in this study may arise from the cell membrane disruption caused by ischemia. Moreover, the disruption of the membrane would subsequently cause cellular contents to leak into the surrounding tissue resulting in an inflammatory response.

Collectively, the progression of ischemic injury has been thought to involve many molecular pathways that play roles in the death of neurons [55]. As shown in Figure 5 cerebral ischemia triggers a complex series of biochemical and molecular disorders that impairs the neurological functions through a breakdown of cellular integrity mediated by energy metabolism, excitotoxic glutamatergic signalling, ionic imbalance, oxidative stress, membrane disruption, osmotic regulation, and so forth [33]. Treatment with HSYA could lead to relative corrections to the changes in these metabolic pathways induced by ischemia. Reversals of metabolic disturbances in the brain after therapy may provide new insights into therapeutic targets for ischemia. 

### 3.4. Inhibition of HSYA on the NF-*κ*B Pathway

**Figure 6:**
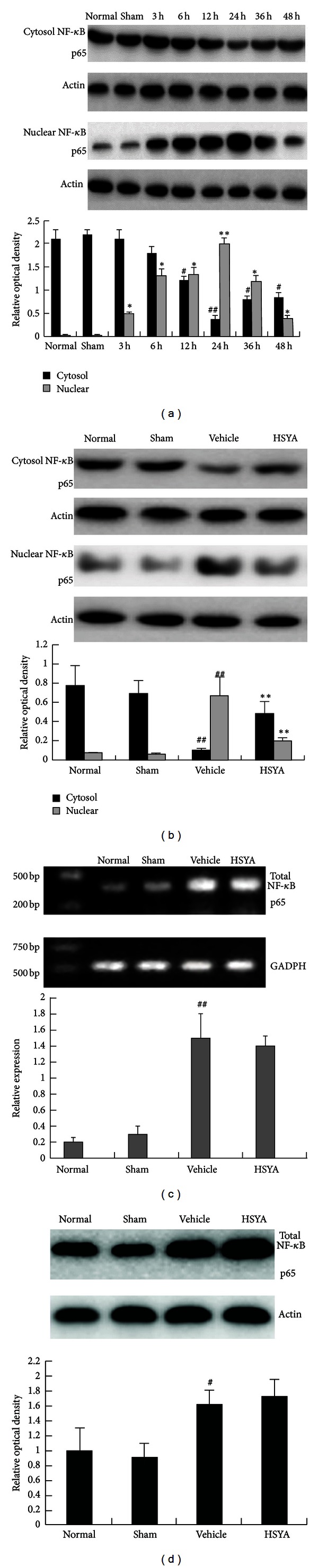
(a) Time course of NF-*κ*B activation, (b) NF-*κ*B p65 activation, (c) total NF-*κ*B p65 mRNA, and (d) protein expression. Data represent $\overset{-}{x}\pm s.\;\text{n}=6$ each group; results represent at least three independent experiments. ^##^ *P* < 0.01 compared with sham group in the cytoplasm or in the nucleus. *P < 0.05, **P < 0.01 compared with model group in the cytoplasm or in the nucleus.

NF-**κ**B activation requires nuclear translocation of the p65 subunit. In this study, the translocation ability of NF-**κ**B p65 subunit from the cytoplasm to the nucleus was investigated by western blotting at different times after pMCAO. As shown in Figure 6(a), expression of the p65 subunit in the cerebral cortex of the ischemic side exhibited an increasing trend in the nucleus. NF-**κ**B was activated as early as 3 h, reached its peak point at 24 h after pMCAO, and then declined. In contrast, p65 expression in the cytoplasm showed the opposite trend; expression reached its lowest point at 24 h. Both in the nucleus and the cytoplasm, NF-**κ**B remained activated at 48 h. These results indicated that NF-**κ**B was activated and translocated from the cytoplasm to the nucleus. Consistent with the infarct volume, the 24 h time point was the most significant (*P* < 0.01) compared to the others (*P* < 0.05). Therefore, the effect of HSYA was evaluated at 24 h after cerebral occlusion in the following experiments.

To determine whether HSYA treatment can interfere with the ischemia-induced activation of the NF-**κ**B p65 subunit, the effect of HSYA on the cytosolic and nuclear pools of p65 protein was evaluated by western blot analysis in the injured ipsilateral cerebral cortex of animals 24 h after artery occlusion. As shown in Figure 6(b), pMCAO considerably increased the nuclear and decreased the cytosolic p65 protein levels. Treatment with HSYA (10 mg/kg/30 min, 5 times) inhibited the increase and decrease of the nuclear and cytosolic p65 levels 24 h after MCAO, respectively. These results suggested that HSYA blocked the nuclear translocation of the p65 from cytoplasm.

**Figure 7:**
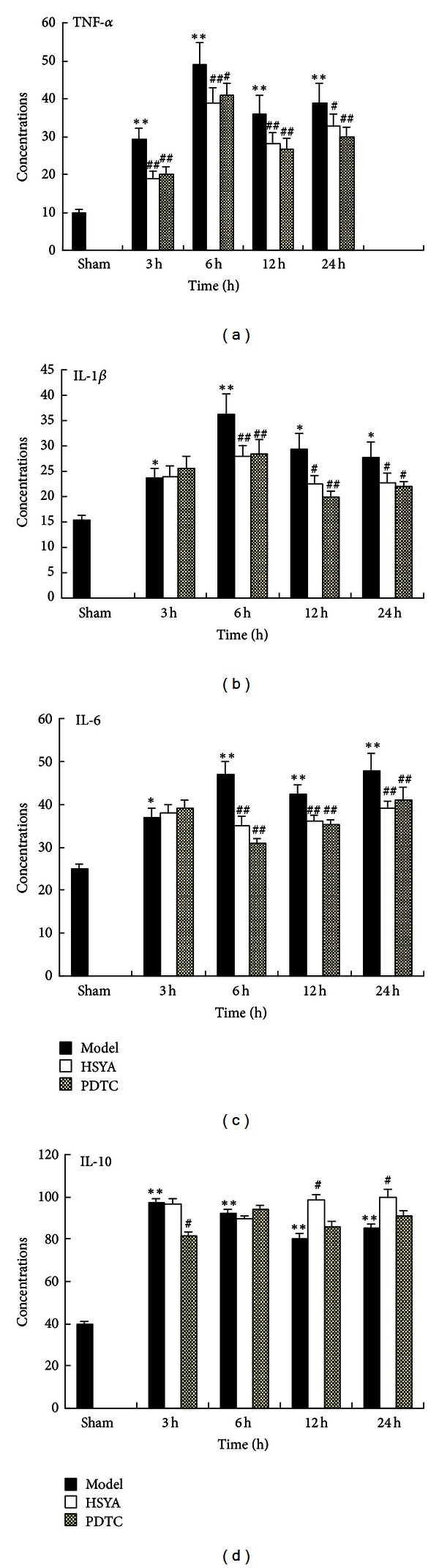
Effect of HSYA on cytokine mRNA expression at 3 h, 6 h, 12 h, and 24 h. (a) TNF-*α* mRNA expression, (b) IL-1*β*mRNA expression, (c) IL-6 mRNA expression, and (d) IL-10 mRNA expression. Data represent $\overset{-}{x}\;\pm \;s$. n = 6 each group; results represent at least three independent experiments. ^##^ *P* < 0.01 compared with sham group. *P < 0.05, **P < 0.01 compared with model group.

Subsequently, we assessed the effect of HSYA on the total p65 mRNA and protein expression by RT-PCR and western blot analysis, respectively. The total p65 mRNA and protein expression levels were increased (Figures 6(c) and 6(d)) during cerebral ischemia, indicating that, 24 h after pMCAO, p65 transcriptional and protein synthesis was induced. However, no effect of HSYA on total p65 mRNA and protein expression was observed. The effect of HSYA on cytokine mRNA expression is displayed in Figure 7.

Activation of NF-**κ**B is due to increased DNA binding of NF-**κ**B after its release from I**κ**B. Because the p65 subunit, which has potent transcriptional activation domains, is known to be important in ischemia [56, 57], we used a p65-specific antibody to assess DNA binding in a microwell colorimetric assay. The NF-**κ**B wild-type and mutated consensus oligonucleotides were used to monitor the specificity of the assay. Wild-type oligonucleotide competed for NF-**κ**B binding to the probe immobilized on the plate, but the mutated consensus oligonucleotide had no effect on NF-**κ**B binding. After 24 h of MCAO, specific NF-**κ**B binding activity was increased in ischemic brains (Figure 2S). When HSYA was continuously administered after ischemia, p65 binding activity decreased by 33.5% (*P* < 0.05). PDTC, the potent NF-**κ**B suppressor was used as a positive control and showed a more significant inhibition of 50.4% (*P* < 0.01).

NF-**κ**B/Rel proteins are transcription factors involved in the activation of an exceptionally large number of genes in response to inflammation, viral and bacterial infections, and other stressful situations requiring rapid reprogramming of gene expression. Stimulation leads to the rapid phosphorylation and, ultimately, proteolytic degradation of I**κ**B, which frees NF-**κ**B to translocate to the nucleus and activate the transcription of its target genes. In the brain, NF-**κ**B regulates the expression of antiapoptotic, proapoptotic, and proinflammatory genes, thereby playing a dual role in neuronal survival [10, 58].

In a few hours after the onset of cerebral ischemia, a multifaceted inflammatory reaction emerges. The transcription of numerous inflammatory mediators is induced [59]. The inflammation contributes to the breakdown of the BBB in cerebral ischemia [60] and is closely interrelated with neuronal cell death, thereby promoting a neurological deficit. The transcription factor NF-**κ**B, as a key regulator of hundreds of genes involved in inflammation, is activated in the hippocampal CA1 neurons after cerebral ischemia [59, 61].

Studies have demonstrated that IL-1*β* and IL-6 genes expressions were induced early following MCAO [62]. TNF-*α* is a deleterious cytokine in stroke [63]. It might activate a cytokine network in the postischemic brain and contribute to an increased sensitivity to and risk of ischemic brain injury [64]. Inhibition of TNF-*α* may represent a novel pharmacological strategy to treat ischemic stroke [65]. Moreover, it is well accepted that IL-10, an anti-inflammatory cytokine, can attenuate brain infarction [66] and provide neuroprotection in ischemic stroke [67]. It is commonly accepted that inhibition of NF-**κ**B will prevent proinflammatory cytokine production, thereby contributing to neuroprotection [68].

In the present study, we noted that HYSA suppressed the translocation of the NF-**κ**B p65 protein from the cytoplasm to the nucleus by western blotting. Additionally, HSYA suppressed p65 DNA-binding activity and the transcription and protein levels of the proinflammatory cytokines TNF-*α*, IL-1*β*, and IL-6. HSYA also promoted both the transcription and protein expression of the anti-inflammatory cytokine IL-10. In the brain, activated NF-**κ**B acted on genes encoding cytokines, cyclooxygenase-2, nitric oxide synthase, and apoptotic proteins [10, 69]. Cerebrovascular inflammation plays a central role in the pathogenesis of cerebral ischemia [70]. Our results suggest that HYSA is also neuroprotective through suppression of the inflammatory NF-**κ**B signaling pathway. This might be one of the mechanisms for the inhibition of apoptosis and protection of cells from ischemic damage. 

Here, we propose neuroprotective mechanisms of HSYA through metabonomics studies combined with an examination of its anti-inflammatory effect through the inhibition of the NF-**κ**B pathway. First, the energy failure or drastic decrease in cellular ATP and glucose levels due to a rapid decrease in glucose uptake following ischemia is responsible for the underlying mechanism of necrosis in the core region [71]. Second, Glu-mediated excitotoxicity could force the effected cell to self-destruct and lead to neuronal damage and eventual cell death. Third, oxidative stress has been shown to activate several intracellular signaling cascades that may have deleterious effects on the cellular homeostasis. One such event leads to activation of MAPKs [71], which are responsible for transmitting extracellular signals into the nucleus. Finally, the changes in the metabolites associated with membrane disruption may cause leakage of cellular contents into the surrounding tissue and invariably a consequent inflammatory response.

## 4. Conclusion

Collectively, cerebral ischemia triggers a complex series of biochemical and molecular mechanism that impairs the neurological functions through breakdown of cellular integrity mediated by energy metabolism, excitotoxic glutamatergic signalling, free-radical reactions, and so forth and then might initiate a series of inflammatory cascades associated with apoptosis or necrosis (Figure 5). On the other hand, NF-**κ**B is a ubiquitously expressed transcription factor that regulates expression of genes involved in inflammation, cell survival, and apoptosis [72, 73]. Our data suggest that HSYA treatment inhibits the NF-**κ**B pathway via suppressing proinflammatory cytokine expression and p65 translocation and binding activity while upregulating an anti-inflammatory cytokine, thereby exhibiting an anti-inflammatory effect, ultimately leading to reduced apoptosis-like cell death. The previous complementary studies suggest that the neuroprotective effect of HSYA is related to the inhibition of neuronal apoptosis or necrosis by modulating inflammatory cascades.

The progression of ischemic injury has been proven to involve many molecular pathways that play roles in the death of neurons. We present new insights into the pathophysiological mechanisms through a systematic integration of histopathological examination, neurological evaluation, BBB permeability studies, metabonomics, and studies of the NF-**κ**B pathway involved in the neuroprotective effect of HSYA after ischemia. The therapeutic effect of HSYA is not a series of independent and isolated metabolic pathways but instead is a complex interconnected network. However, certain limitations exist in the present studies. How does HSYA travel across the BBB? We have detailed an effective method for detecting HSYA in brain samples and verified that HSYA could cross the BBB of ischemic and normal rats. As such, the mechanism of how HSYA, a small, water-soluble compound, can cross the BBB and whether its neuroprotective effect can be enhanced by an improvement in BBB permeability and integrity still needs to be investigated in future. The duration of ischemia is an important prognosis determinant. Reperfusion also plays a prominent role in damage distribution and is responsible for oxidative stress due to the generation of free radicals [57]. The reperfusion-induced inflammatory infarction is much more harmful and complicated than ischemia. In the present study, a series of metabolic pathways associated with inflammation were found during brain ischemia. In the future, changes in the stage of reperfusion and the precise mechanisms of how HSYA protects against focal cerebral ischemia need to be further investigated.

## Supplementary Material

Table 1S: Sequence of primers.
Table 2S: Neurological deficit scores of different administrations of HSYA were observed in vehicle-treated MCAO rats.
Table 3S: ^1^H NMR Chemical shifts of main metabolites contributing to the classification of ischemia in different brain tissues.
Figure 1S: Loading plots from the OPLS-DDA of the NMR spectra of (a)–(b) cerebellum, (c)–(d) cortex and (e)–(f) hippocampus. Note the most similar metabolic changes that occurred within these tissues following ischemia. These loading plots corresponded to the scores plots in Fig. 4 (a), ((b), (d), (e), (g) and (h), respectively. Positive peaks corresponded to metabolites that were at higher concentration in ischemia animals and were assigned as described in the legend to Figure 3.
Figure 2S: Effect of HSYA and PDTC on NF-**κ**B binding activity after pMCAO. Data represent *x* ± *s* . n = 6 each group; results represent at least three independent experiments. ## p<0.01 compared with sham group. ∗ p 0.05, ∗∗ p<0.01 compared with model group.

## Abbreviations

HSYA: Hydroxysafflor yellow A
BBB: Blood-brain barrier
NF-*κ*B: Nuclear factor-*κ*B
pMCAO: Permanent middle cerebral artery occlusion
SFDA: State Food and Drug Administration
NMR: Nuclear magnetic resonance spectroscopy
HPLC-MS^n^: High-performance liquid chromatography coupled with multistage tandem mass spectrometry
HR MAS: High-resolution magic-angle spinning
PDTC: Pyrrolidine dithiocarbamate ammonium salt
TSP: 3-trimethlsilyl-2,2,3,3-d_4_-propionate
CPMG: Carr-Purcell-Meiboom-Gill
OPLS-DA: Orthogonal partial least-squares discriminant analysis
TTC: 2,3,5-triphenyl tetrazolium chloride
GAPDH: Glyceraldehyde-3-phosphate dehydrogenase
CNS: Central nervous system
Lac: Lactate
Ala: Alanine
GABA: Gamma-aminobutyric acid
Ace: Acetate
NAA: *N*-acetyl aspartate
Glu: Glutamate
Gln: Glutamine
Asp: Aspartate
Cre: Creatine
Cho: Choline
Pcho: Phosphocholine
Tau: Taurine
Myo: Myo-inositol.
