# Supplementary material for: A Systematic, Integrated Study on the Neuroprotective Effects of Hydroxysafflor Yellow A Revealed by 1H NMR-Based Metabonomics and the NF-κB Pathway
Source: Evid Based Complement Alternat Med. 2013 Apr 22;2013:147362. doi: 10.1155/2013/147362 (PMC3654365; doi:10.1155/2013/147362)
Supplement: Supplementary file 1 — Table 1S: Sequence of primers. Table 2S: Neurological deficit scores of different administrations of HSYA were observed in vehicle-treated MCAO rats. Table 3S: 1H NMR Chemical shifts of main metabolites contributing to the classification of ischemia in different brain tissues. Figure 1S: Loading plots from the OPLS-DDA of the NMR spectra of (a)–(b) cerebellum, (c)–(d) cortex and (e)–(f) hippocampus. Note the most similar metabolic changes that occurred within these tissues following ischemia. These loading plots corresponded to the scores plots in Fig. 4 (a), ((b), (d), (e), (g) and (h), respectively. Positive peaks corresponded to metabolites that were at higher concentration in ischemia animals and were assigned as described in the legend to Figure 3. Figure 2S: Effect of HSYA and PDTC on NF-κB binding activity after pMCAO. Data represent x ± s . n = 6 each group; results represent at least three independent experiments. ## p<0.01 compared with sham group. ∗ p 0.05, ∗∗ p<0.01 compared with model group. [file 147362.f1.pdf]

Supporting information

A systematic, integrated study on the neuroprotective effects of hydroxysafflor yellow A revealed by ^{1}H NMR-based metabonomics and the NF-κB pathway

Yuanyan Liu [1,2#], Zeqin Lian[1#], Yinghong Wang[1], Shishan Yu[1*], Haibo Zhu[1*], Tingting Chen[1], Jing Qu[1], Jianbei Li[1], Shuangang Ma[1] and Xianhong Chen[1]*

[1] State Key Laboratory of Bioactive Substance and Function of Natural Medicines, Institute of Materia Medica, Chinese Academy of Medical Sciences and Peking Union Medical College, Beijing 100050, China

[2] School of Chinese Materia Medica, Beijing University of Chinese Medicine, Beijing, 100102, China

[*] Corresponding author

[#] These authors contributed equally to this work.

Email addresses:

YYL: yyliu_1980@hotmail.com

ZQL: popular@imm.ac.cn

YHW: wyh@imm.ac.cn

SSY: yushishan@imm.ac.cn

HBZ: zhuhaibo@imm.ac.cn

TTC: qukai@imm.ac.cn

JQ: qujing@imm.ac.cn

JBL: lijianbei@imm.ac.cn

SGM: shuanggang2008@126.com

XHC: xiaotian18240@yahoo.com.cn

Materials and Methods

MCAO model construction

The rats underwent permanent MCAO as previously described.[1] Briefly, rats were anesthetized with 2.0% isoflurane in 30% oxygen and 70% nitrous oxide with rectal temperature controlled at 37 ℃ using a homoeothermic pad. The right common carotid artery (CCA), external carotid artery (ECA) and internal carotid artery (ICA) were isolated. An 18-20 mm length of nylon suture (φ: 0.24 mm) with its tip rounded by gentle heating was introduced into the ECA lumen and advanced into the ICA to block the origin of the middle cerebral artery. The caudal tail artery was cannulated for continuous monitoring of physiological variables (mean arterial blood pressure, arterial blood gases, and blood glucose) level. The rats recovered completely from the anesthesia before returning to the cages with free access to food and water. The sham operated rats received all surgical procedures but without the suture inserted. Rigorous room temperature control (22 ± 2 ℃) was performed post surgery.

1. Longa, E. Z.; Weinstein, P. R.; Carlson, S.; Cummins, R., Reversible middle cerebral artery occlusion without craniectomy in rats. *Stroke* **1989,** *20* (1), 84-91.

Table 1S Sequence of primers

mRNA	Sequence	Cycle	Tm/◦C	Product/bp
**TNF-*α***	F: 5’- TGTCTACTGAACTTCGGGGTGATC -3’	35	56	348
	R：5’- CTGGTATGAAATGGCAAATCGG -3’			
**IL-1*β***	F：5’-AATGACCTGTTCTTTGAGGCTGAC-3’	35	56	408
	R：5’-TTGGCTTATGTTCTGTCCATTGAG-3’			
**IL-6**	F: 5’- CTTCTTGGGACTGATGTTGTTGAC -3’	35	56	395
	R：5’- TGCTCTGAATGACTCTGGCTTTG -3’			
**IL-10**	F：5’-GCTATGTTGCCTGCTCTTACTGG-3’	35	56	403
	R：5’-CTCCACTGCCTTGCTTTTATTCTC-3’			
**GAPDH**	F：5'-ACCACCATGGAGAAGGCTGG-3’	23	62	528
	R：5'-CTCAGTGTAGCCCAGGATGC-3’			

Table 2S Neurological deficit scores of different administrations of HSYA were observed in vehicle-treated MCAO rats.

	Neurological score
Normal	0
Sham	0
Vehicle	8.16±0.75$^{\#\#}$
HSYA (pre-10mg/kg)	6.70±1.18*
HSYA (post-10mg/kg)	6.80±1.32*
HSYA (post-50mg/kg)	6.70±0.78*
HSYA (10mg/kg/30min, 5 times)	6.00±0.89**

Normal group: n=6; the other group: n=10

$^{\#\#}p<0.01$ *vs* Sham; $^{*}p<0.05$, $^{**}p<0.01$ *vs* Vehicle

Table 3S ^{1}H NMR Chemical shifts of main metabolites contributing to the classification of ischemia in different brain tissues.

**NO.**	**Metabolite**	**abbreviation**	**Chemical shifts (ppm)**	***P < 0.05***
1	Lactate	Lac	1.33, 4.12	↑
2	Alanine	Ala	1.48, 3.79	↑
3	Acetate	Ace	1.92	↑
4	Aspartate	Asp	2.68, 2.82	↑
5	Gamma aminobutyric acid	GABA	1.91, 2.30, 3.02	↓
6	N-acetyl aspartate	NAA	2.03, 2.51, 2.70, 4.40	↓
7	Glutamate	Glu	2.07, 2.36, 3.77	↑
8	Glutamate/Glutamine	Glu/Gln	2.07, 2.36, 2.14, 2.46, 3.77	↑
9	Creatine	Cre	3.04, 3.93	↓(cerebellum, cortex); ↑(hippocampus)
10	Choline	Cho	3.19, 3.52, 4.07	↑
11	Phosphocholine	Pcho	3.21, 3.52, 4.07	↑
12	Taurine	Tau	3.25, 3.43	↓
13	Myo-insitol	Myo	3.28, 3.56, 3.63, 4.06	↓

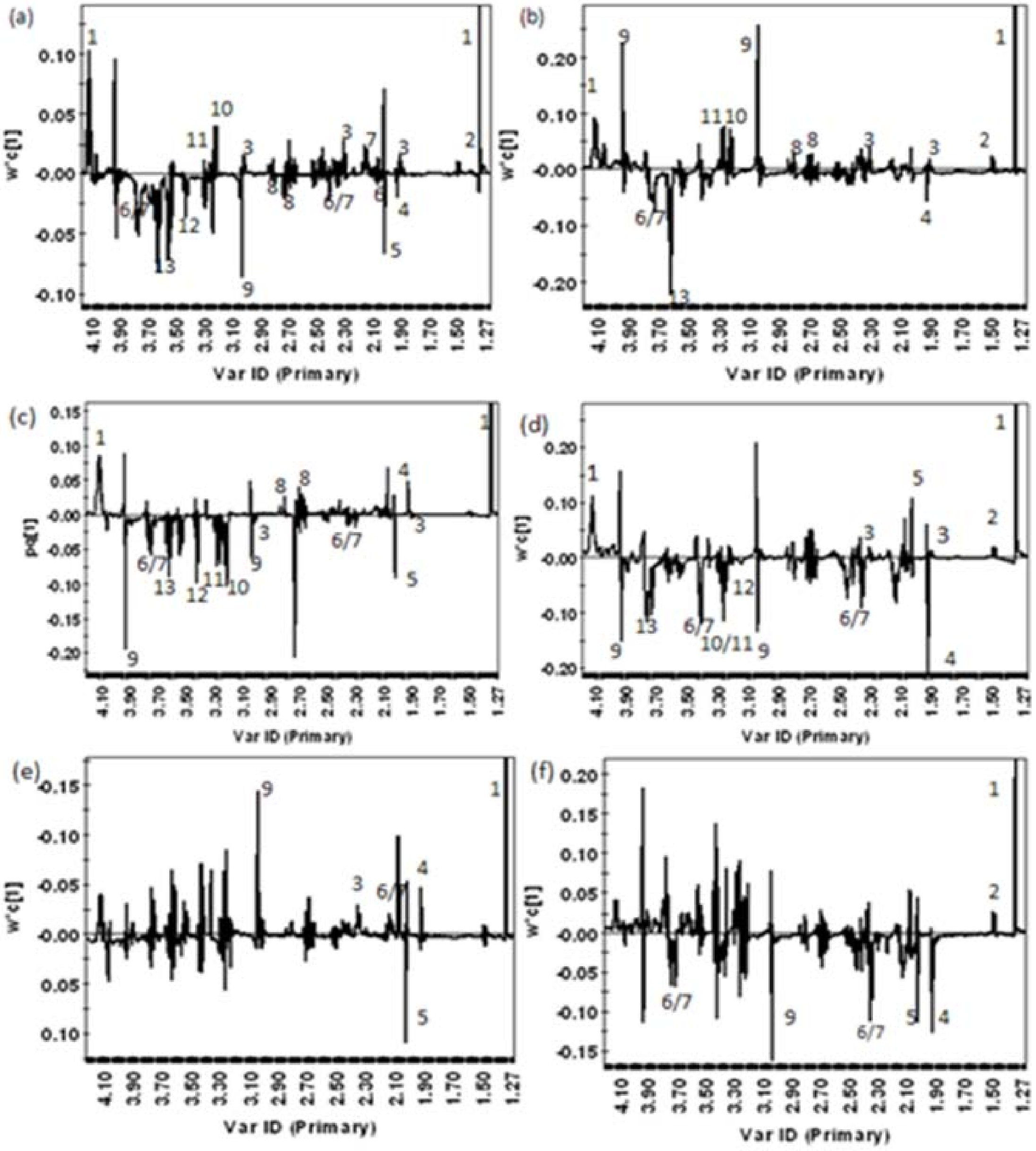

Figure 1S. Loading plots from the OPLS-DA of the NMR spectra of (a)–(b) cerebellum, (c)–(d) cortex and (e)–(f) hippocampus. Note the most similar metabolic changes that occurred within these tissues following ischemia. These loading plots corresponded to the scores plots in Fig. 4 (a), (b), (d), (e), (g) and (h), respectively. Positive peaks corresponded to metabolites that were at higher concentration in ischemia animals and were assigned as described in the legend to Fig. 3.

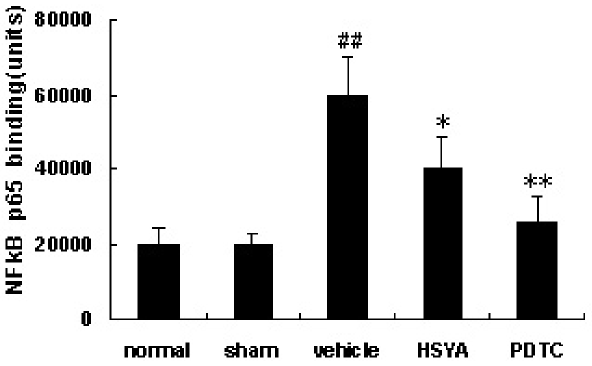

Figure 2S. **Effect of HSYA and PDTC on** NF-*κ*B binding activity after pMCAO. Data represent $\bar{x} \pm s$. n=6 each group; results represent at least three independent experiments. $^{\#\#}$ p<0.01 compared with sham group. * p<0.05, ** p<0.01 compared with model group.
